# New Methods of Enhancing the Thermal Durability of Silica Optical Fibers

**DOI:** 10.3390/ma7106947

**Published:** 2014-10-13

**Authors:** Karol Wysokiński, Tomasz Stańczyk, Katarzyna Gibała, Tadeusz Tenderenda, Anna Ziołowicz, Mateusz Słowikowski, Małgorzata Broczkowska, Tomasz Nasiłowski

**Affiliations:** 1 InPhoTech, ul. Slomińskiego 17/31, 00-195 Warszawa, Poland; E-Mails: tstanczyk@inphotech.pl (T.S.); ttenderenda@inphotech.pl (T.T.); tnasilowski@inphotech.pl (T.N.); 2 P.H. ELMAT, Rogoźnica 312, 36-060 Głogów Małopolski, Poland; E-Mail: katarzynagibala@interia.eu; 3 Institute of Applied Physics, Military University of Technology, ul. Kaliskiego 2, 00-980 Warszawa, Poland; 4 Polish Centre for Photonics and Fibre Optics, Rogoźnica 312, 36-060 Głogów Małopolski, Poland; E-Mails: aziolowicz@pcfs.org.pl (A.Z.); mslowikowski@pcfs.org.pl (M.S.); mbroczkowska@pcfs.org.pl (M.B.)

**Keywords:** optical fiber sensors, harsh environments, microstructured optical fibers, metal-coated optical fibers, thermal degradation of silica, thermal reliability of optical fibers

## Abstract

Microstructured optical fibers can be precisely tailored for many different applications, out of which sensing has been found to be particularly interesting. However, placing silica optical fiber sensors in harsh environments results in their quick destruction as a result of the hydrolysis process. In this paper, the degradation mechanism of bare and metal-coated optical fibers at high temperatures under longitudinal strain has been determined by detailed analysis of the thermal behavior of silica and metals, like copper and nickel. We furthermore propose a novel method of enhancing the lifetime of optical fibers by the deposition of electroless nickel-phosphorous alloy in a low-temperature chemical process. The best results were obtained for a coating comprising an inner layer of copper and outer layer of low phosphorous nickel. Lifetime values obtained during the annealing experiments were extrapolated to other temperatures by a dedicated model elaborated by the authors. The estimated copper-coated optical fiber lifetime under cycled longitudinal strain reached 31 h at 450 °C.

## 1. Introduction

Optical fibers have revolutionized the telecommunication industry, due to their remarkable properties, such as, for example, large data transfer rate, possibility of multiplexing many signals in one fiber and immunity to external conditions [1]. Furthermore, in the last three decades, optical fibers were widely investigated for applications in the sensing industry. This is mainly because of their compact size, flexibility, possibility of both point and distribution sensing and often low cost [2]. Furthermore, no electric current is needed for their operation, which together with their temperature performance (compared to existing electronic solutions), makes them perfect for sensing in explosion endangered zones.

Microstructured optical fibers have all of the advantages of standard optical fiber, and additionally, by the possibility of modifying the air-hole microstructure, they offer an additional degree of freedom in fiber design, making it possible to fabricate fibers dedicated for specific sensing applications (*i.e.*, pressure, curvature, temperature, strain, torsion, vibration, gas and liquid, as well as electric and magnetic fields sensing [3]). What is more, similarly to traditional fibers, there are also many different methods of sensing with microstructured fibers, which include, e.g., polarization changes [4,5], change of refractive index [6], interference [7] or fiber Bragg gratings [8]. Apart from the physical parameters, the concentration of chemical and biochemical entities can also be detected using microstructured optical fibers. This includes sensors of pH [9], humidity [10], gases, such as methane [11] or ammonia, and even biochemical sensors, which can detect molecules, like proteins [12] or DNA [13]. The principle of operation of chemical sensors can be based on absorption within a hollow core [11], evanescent field absorption [14], fluorescence [15] or Raman spectroscopy (including surface-enhanced Raman spectroscopy [16]).

Optical fibers intended for use at high temperatures need to be coated with a thick layer of metal, which will separate the fibers from the external environment. This is crucial due to the degradation of silica in such conditions, which results in a severe deterioration of the mechanical parameters. However, in extreme environments (*i.e.*, temperatures reaching 500 °C), the most frequently used metal coatings oxidize and leave the surface of the fiber unprotected. This may not be a major issue in steady-state high temperature tests; however, in the majority of industrial applications, fibers are frequently stretched or bent, which is caused by manifold factors, like, for example, thermal expansion of the sensor case. Therefore, it is important to perform long-term experiments under strain, because they will better correspond to the real-life operation of optical fiber devices.

In this work, we present our approach to deposit high-temperature-resistant metal on optical fibers by a low temperature chemical method. We furthermore examine the impact of a high-temperature conditioning on mechanical parameters of bare silica fibers and their metal coatings. Sample fibers were annealed for a long time at high temperatures, and after each hour, they were stretched to see the influence of both strain and temperature. Additionally, since temperature reliability tests are time consuming, we have elaborated a model enabling one to extrapolate the obtained results to other temperatures.

## 2. Degradation of Optical Fibers at High Temperature

### 2.1. Degradation of Silica

Fiber optic sensors used at temperatures exceeding 350 °C cannot be embedded in a polymer coating, due to the thermal degradation of plastics in such conditions [17]. Silica is well known for its inertness at low temperatures and is a valuable material for laboratory equipment. Nevertheless, SiO_2_ undergoes the reaction of hydrolysis, resulting in a formation of Si–O–H silanol groups [18], according to Equations (1) and (2):

(1) $${\text{H}}_{\text{2}}{\text{PO}}_{\text{2}}^{\text{–}}+{\text{H}}_{\text{2}}\text{O}\to {\text{H}}_{\text{2}}{\text{PO}}_{\text{3}}^{\text{–}}+2{\text{H}}^{\text{+}}+2e^{\text{–}}$$

(2) $$\equiv \text{Si–O–Si}\equiv {\text{ + OH}}^{\text{–}}\to \;\equiv {\text{Si–O}}^{\text{–}}\text{+ HO–Si}\equiv$$

The latter equation proves that alkaline solutions are particularly aggressive due to the strong corrosive properties of OH^−^ ions [19]. The hydrolysis reaction worsens the mechanical parameters of fibers, because of the presence of cracks and flaws on the fiber surface. It was shown that high air humidity has a strong negative impact on silica fiber bending strength [20]. In regard to aging and fatigue, it was also proven that the increase of temperature with constant relative humidity causes the deterioration of mechanical properties [21]. This can be attributed to a higher reaction rate at higher temperatures, since the reaction rate constant usually is given by the Arrhenius expression. Likewise, the most popular optical fiber fatigue models employ typical chemical reaction dependencies [20,21]. It was shown that the silica hydrolysis reaction order was two with respect to the air humidity [21]. All of the fatigue models assume that water reacts with silica, which produces fractures on the fiber surface, which finally worsen its mechanical parameters. This is an intuitive postulate, since it is well known that even a small crack on a rigid object becomes a weak point, at which breakage is likely to occur. This property is widely used in the cleaving of optical fibers, glass capillaries, ceramic tiles, *etc.*

### 2.2. Metal Coatings and Their Oxidation at High Temperatures

Considering the issues mentioned above, using bare optical fibers is not a recommended choice for high temperature applications. This is why metal-coated fibers are used for such conditions instead [22]. The deposition of metals onto the fiber is usually performed by an adaptation of the Ohno continuous casting process [22,23]. This process is based on drawing the fiber through a liquid metal near its melting point, which enables molten metal to solidify on the fiber surface. This means that the choice of possible metal coatings is limited to those with a relatively low melting point. Therefore, the most popular and commercially available coating materials are copper, aluminum and gold. While gold coated fibers are the least corrosion susceptible, their high price makes them also the least interesting for commercial applications. Furthermore, aluminum has a low melting point (660 °C for pure metal), which excludes it from extreme temperature applications. On the other hand, copper, with its melting point of above 1000 °C and relatively low price, seems to be the best candidate for high temperature applications. However, the high oxidation rate of copper is a factor that should be taken into account.

After heating copper-coated fibers to temperatures exceeding 500 °C in air, the metal coating changes its color to black, and the fiber becomes extremely brittle. Three distinct temperature ranges of copper oxidation kinetics can be distinguished [24]: 350–550 °C, 550–850 °C and 850–1050 °C. They all differ in the mechanism and oxidation rate. In general, the mechanism of the oxidation process is based on the outward diffusion of copper atoms from the metal substrate to the surface [24]. Long exposure to an oxidizing atmosphere produces cracks within the oxide layer [24], which finally becomes porous [25]. Both the porosity of the oxide layer and the diffusion of copper atoms to the surface contribute to the destruction of copper-coated fibers. Oxygen reacts with the metal layer to produce copper oxides. As the thickness of the oxide layer grows, cracks and pores can be formed on its surface. Eventually, the porosity of this layer enables aggressive agents, like water, to reach the fiber surface and develop cracks, which finally results in the deterioration of the mechanical properties of a fiber.

### 2.3. Enhancing the High Temperature Performance of the Optical Fibers

Surface modification can prevent the inevitable destruction of copper-coated optical fibers. The most straightforward approach could be an electrochemical deposition of a thin noble metal layer. However, copper exhibits a high interdiffusion rate with precious metals, like gold, platinum or palladium [26,27], which makes it impossible to use such materials without a suitable middle layer acting as a diffusion barrier [26]. Another way is to deposit thin (approximately 1 μm) films of pure nickel, chromium or electroless Ni–P alloy, which were reported to provide good results for blocking the diffusion of copper, even at elevated temperatures up to 550 °C [27]. What, however, needs to be taken into account is that nickel and copper are mutually soluble at higher temperatures of the order of magnitude of 750 °C [26]; therefore, the nickel layer needs to be relatively thick to work in such conditions. It is even possible to plate bare silica optical fiber with nickel (with an appropriate underlayer), which omits the problems related to susceptibility to corrosion of copper [28], but it is technologically challenging to use this method to deposit metals on longer (e.g., 1 m) fragments of optical fibers or on more complex sensing transducers. There are also other methods of enhancing the corrosion resistance of copper, like chromate conversion coating or coloring, already known for decades. However, all of the compounds that usually build these layers are not thermally resistant. For instance, the black layer of copper sulfide oxidizes in air at 500 °C, and a synthetic patina can be unstable even at room temperature. 

Since the deposition of nickel seems to be the best means to improve the copper-coating resistance to high temperatures, we verify this approach in our work. There are two main methods of nickel plating—electrolytic and electroless. In the electrolytic method, nickel is reduced from a nickel salt solution by electrolysis; whereas the electroless method employs a chemical reagent to reduce nickel ions from the solution to the metallic form. The major advantages of the electrolytic method are: the possibility of reaching high deposition rates, the fact that the product is a chemically pure metal and the opportunity of tuning the mechanical parameters by solution composition and current density. The biggest disadvantage of this method is the uneven distribution of the coating thickness, dependent on the shape of the coated object. This property is not a major issue for optical fibers, since it is possible to arrange the anodes in an appropriate way around the fiber. However, when a more complex object (e.g., sensor head consisting of a fiber and a transducer) is to be coated, achieving a uniform distribution of electric field becomes extremely difficult. In the electroless method, this problem is not encountered, because the reduction reaction takes place on the entire activated surface; hence, even very sophisticated surfaces may be uniformly coated. Along with the additional possibility of the deposition of nickel on non-conductive surfaces, the electroless plating process becomes a very attractive method of enhancing the high temperature resistance of both bare silica and copper-coated optical fibers, as well as more complex fiber components. 

### 2.4. Electroless Nickel Plating and the Preparation of Samples

For a proper operation, the electroless method requires three basic components: a metal salt, a reducing agent and a catalyst. Nickel plating solutions, obviously, must contain Ni^2+^ ions; however, presence of other metals can yield the formation of alloys. The reducing agent reduces nickel ions to the metal form and simultaneously is being oxidized, according to Equation (3) (the reaction takes place only at the surface of a catalyst):

(3) $${\text{Ni}}^{\text{2+}}\text{+ red}\to \text{Ni + ox}$$

The electroless plating method is different from other chemical reduction methods, because the deposited metal is a catalyst of the reaction. This means that once a proper catalyst is deposited at the surface of an object, the metal layer will be continuously growing in an electroless plating bath. Eventually, thick layers of metal up to 1000 μm can be obtained [29].

It is believed that 99% of the world’s electroless nickel was being plated using sodium hypophosphite as the reducing agent [30]. Reaction Equation (4) describes such a process:

(4) $${\text{Ni}}^{\text{2+}}+{\text{4H}}_{\text{2}}{\text{PO}}_{\text{2}}^{\text{–}}+{\text{H}}_{\text{2}}\text{O}\to \text{Ni}+\text{P}+{\text{2HPO}}_{\text{3}}^{\text{2–}}+{\text{H}}_{\text{2}}{\text{PO}}_{\text{3}}^{\text{–}}+{\text{3H}}^{\text{+}}+{\text{3/2H}}_{\text{2}}$$

According to Brennel and Riddell, this can be divided into several steps [30]. The first one is an anodic reaction of water and hypophosphite:

(5) $${\text{H}}_{\text{2}}{\text{PO}}_{\text{2}}^{\text{–}}+{\text{H}}_{\text{2}}\text{O}\to {\text{H}}_{\text{2}}{\text{PO}}_{\text{3}}^{\text{–}}+2{\text{H}}^{\text{+}}+2e^{\text{–}}$$

At the same time, three distinct cathodic reactions utilizing electrons released in the latter process can occur:

(6) $${\text{Ni}}^{\text{2+}}+2e^{\text{–}}\to \text{Ni}$$

(7) $$2{\text{H}}^{\text{+}}+2e^{\text{–}}\to {\text{H}}_{\text{2}}$$

(8) $${\text{H}}_{\text{2}}{\text{PO}}_{\text{2}}^{\text{–}}+2{\text{H}}^{\text{+}}+e^{\text{–}}\to \text{P}+2{\text{H}}_{\text{2}}\text{O}$$

The last equation shows that apart from the Ni metal, also P is formed during the plating process. Phosphorous is incorporated into the nickel layer forming Ni–P alloy. These equations also explain the influence of pH on the coating composition. The content of phosphorous usually resides within 2%–13% [30]; however, lower and higher concentrations can also be achieved. It is noteworthy that there are also other equivalent mechanisms describing the process of electroless nickel plating [31].

As stated above, electroless plating requires in its initial stage the presence of an appropriate catalyst. It is known that the most suitable ones are the noble metals, with palladium providing the highest plating rates [32]. The deposition of Pd on a copper substrate can be performed simply by dipping a fiber in a water solution containing PdCl_2_ and HCl for a few minutes.

Electroless nickel can be deposited not only on metal substrates like Cu, but also on insulators. This approach can possibly lead to omitting the problem of the copper oxidation and deposition directly on bare silica fiber. Nevertheless, more sophisticated methods of the substrate pre-treatment are required.

The properties of electroless Ni alloy are strongly dependent on its phosphorous content. Corrosion resistance changes along with P percentage [30], e.g., low phosphorous alloys are resistant to high temperatures and alkali solutions, but they are prone to acids, whereas high phosphorous alloys are acid-resistant, but corrode in alkali environments and oxidize quicker at high temperatures, due to the oxidation of P. From the mechanical point of view, the most important properties of the metal layer are: hardness, ductility and internal stress. As the strong mechanical wear of the fibers in fiber optics is not commonly encountered, the best alloy for such applications should have low hardness and high ductility. This is evident in high-phosphorous samples [30]; however, high temperature conditioning causes a rapid increase of the hardness of such a layer. Internal stress should be as low as possible, and this is encountered in medium-phosphorous (*ca*. 10% P) alloy. However, the same alloy has a very low ductility, which can lead to the evolution of cracks in the coating during bending. On the other hand, low-phosphorous deposits have high hardness, moderate ductility and high internal stress [30]. What is more, after annealing, the properties of electroless nickel can change, as it becomes harder and more brittle [33]. All of these factors show that it is necessary to optimize the P content in the alloy to obtain coatings with the best performance at high temperatures. In our work, we experimentally evaluated three different nickel-phosphorous alloys in order to determine which gives the best results during long-term exposure to high temperatures.

## 3. Experiments and Results

### 3.1. Sample Preparation

Electroless nickel was deposited on approximately 1-m long copper-coated optical fibers (sole or attached to a metal membrane) according to a procedure consisting of the following steps: degreasing in NaOH, etching in HNO_3_, activation in PdCl_2_/HCl solution and electroless plating. Each step was followed by rinsing. Solution composition and plating principles can be found in [31].

Firstly, we have deposited a thin, approximately 2 μm, layer of nickel alloys with low (3.5%), medium (10%) and high (13%) phosphorous content on commercially available copper-coated fibers. The amount of phosphorous in the deposit was controlled by adjustment the pH and the concentrations of nickel and hypophosphite ions. Usually, the amount of Ni^2+^ and H_2_PO_2_^−^ is relatively high, which is convenient for the long-term use of the plating bath, because above a certain level of concentrations, the deposit composition is constant (it does not change as the reduction reaction takes place). This means that the crucial variable for P percentage is the pH value for a given type of bath. Alkaline solutions give low P content, while weakly acidic baths give a medium amount of P, whereas more acidic solutions give a high phosphorous alloy. 

As presented in the following paragraphs, annealing at 600 °C indicated that medium-phosphorous samples were much more fragile than the two other alloys. Subsequently, approximately 5-μm thick layers of electroless nickel (high and low P) were deposited on two copper-coated fiber samples, which were then examined for longitudinal strain resistance at high temperatures of 600–700 °C. In the next step, a 5-μm layer of low-phosphorous nickel was deposited on a copper membrane (which can be used as sensor transducer [34]) with copper-coated fibers electrochemically bonded to them. For the minimum bending radius tests, a 10-μm layer of low-phosphorous electroless nickel was examined.

### 3.2. Tensile Strength of Copper Coated Fibers at Room Temperature

For the first set of tests, we used common single mode fibers coated with a 20-μm layer of copper, which in terms of high temperature degradation, can be considered an equivalent of any type of silica optical fiber (e.g., microstructured, polarization maintaining, *etc.*). Along with the standard copper-coated fibers, we have also examined more complex fiber optic transducers (*i.e.*, copper-coated fibers electrolytically bonded to a metal membrane [34]). The tensile tests were performed on a linear translation stage, to which the fibers and copper membranes were attached.

**Figure 1:**
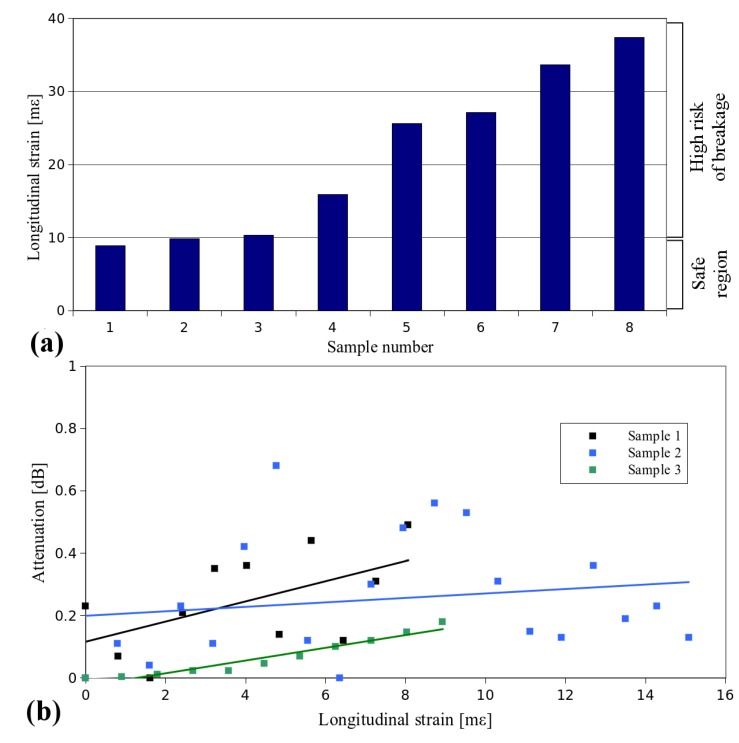
(**a**) Tensile strength of 8 copper-coated optical fibers with longitudinal strain values at breakage given on the *y*-axis. (**b**) Attenuation as a function of longitudinal strain.

The result of the tensile strength measurement is a maximum relative elongation (*i.e.*, longitudinal strain) at which the sample breaks. What needs to be noted is that during the tensile strength tests, a breakage always occurred on the fiber itself and not at the membrane-to-fiber joint, which proves the high strength of such a connection and the fact that the tensile strength of the fiber is more critical. Our first goal was to check the safe value of strain that can be applied to a copper coated fiber without breaking it. Taking into account the strength of 8 measured samples (Figure 1a), we have set the safe longitudinal strain for the subsequent tests below 10 mε. The low tensile strength of copper-coated fibers, together with the relatively wide spread of results (Figure 1a), in comparison to standard polymer-coated fibers (with reported elongations of 6.5% at rupture [35]) can be attributed to the strain introduced to the fiber during high temperature processing (*i.e*., deposition of copper). Therefore, not only the longitudinal strain, but also the annealing time should be considered during the analysis of the further experiments.

Subsequently, we have measured the transmission during mechanical stretching. The results shown in Figure 1b indicate that there is no significant difference in the intensity of the transmitted light until the sample breaks, which means that such optical fibers can be used in sensing applications, in which the fiber is subjected to continuous longitudinal strain without any characteristic decay in the fiber transmission.

### 3.3. High Temperature Annealing of Metal-Coated Fibers

#### 3.3.1. Annealing of Copper Coated Optical Fibers

**Figure 2:**
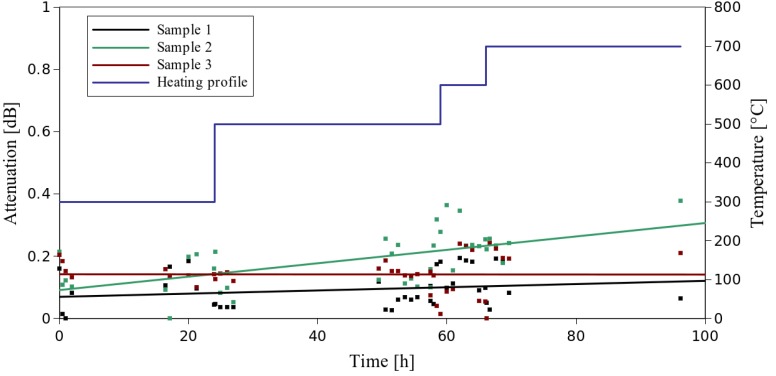
Heating profile and attenuation change over the time of measurement of copper-coated fibers.

As temperature is the most critical factor decreasing the reliability of copper coated fibers, we have performed high temperature annealing tests in a furnace. The applied temperature was modified and increased during the test in order to speed up the process of degradation (with the temperature profile depicted in Figure 2 as a blue line). Furthermore, the fiber samples were coupled with a VCSEL (Vertical-Cavity Surface-Emitting Laser) source and a power meter in order to monitor changes in transmission during the heating process (with the measurement density increased at the temperature transitions). As one can see in Figure 2, no significant change in the fiber transmission can be noticed until the end of the process, after over 70 h of annealing at temperatures of 500 °C and higher. What was, however, observed was the extreme decay of the mechanical resistance of the samples. The copper surface changed its color to black, and the fibers became extremely brittle (which was related to the copper surface oxidation process). No manual operation was then possible, making the samples useless for further operation, since even a small strain applied to a fiber resulted in a breakage.

Taking into account real-life sensor operation conditions, at which the fiber may be simultaneously subjected to strain and high temperature, we propose an additional test of strain after annealing, in which the samples are taken out of the furnace oven every hour and exposed to strain at room temperature. The longitudinal strain was applied to the samples gradually until reaching the maximum value of 7.5 mε (according to the results presented in Section 3.1). Such a test allowed one to check *in situ* the condition of the fiber and simulate real life sensor operation under maximum strains.

#### 3.3.2. High Temperature Durability of Repeatedly Stretched Copper-Coated Optical Fibers

Evaluation of the electroless nickel influence on the optical fiber high temperature performance should be preceded by checking the properties of the optical fibers only coated with copper. We have annealed 20 cm-long samples of copper-coated fibers at different temperatures, and after every hour (±1 min) of annealing, they have been stretched to a value of 7.5 mε. The results of the measurements are presented in Table 1.

**Table 1:** The results of the tensile stress experiments performed every hour during annealing at varied temperatures. All of the samples were Cu-coated optical fibers, which were repeatedly stretched every hour. Each value represents the time of annealing, after which the sample broke.

Sample Number	Hours at 500 °C	Hours at 550 °C	Hours at 600 °C	Hours at 650 °C
1	21	–	–	–
2	–	15	–	–
3	–	15	–	–
4	–	–	9	–
5	–	–	–	5
6	–	–	–	5

These results show that the time of life of the copper-coated fibers decreases non-linearly while the temperature increases. Such a test gives plenty of information about the destruction kinetics. Further work, including a detailed analysis of these results and an extrapolation model, can be found in Section 4.

#### 3.3.3. Tensile Strength Measurements of Annealed Metal-Coated Fibers

Initial annealing experiments conducted with a thin, approximately 2 μm, layer of electroless nickel indicated that low and high phosphorous coatings endure longer at high temperatures than medium phosphorous deposits. Therefore, we have tested two samples of copper-coated fibers with an overlayer of low and high-phosphorous nickel (with an additional two samples coated only with copper for reference). The thickness of the coating was approximately 5 μm. During every experiment, samples were stretched after each hour of annealing. The tensile strain was applied to the fiber gradually with a step of approximately 0.05 mε up to approximately 7.5 mε.

The results are presented in Table 2. It can be seen that nickel coated fibers (5 μm of Ni) are more resistant to high temperature, since they endure one hour longer at 700 °C. Furthermore, the low-phosphorous alloys had a higher value of tensile strength at breakage. Low P deposits exhibit a lower oxidation rate at high temperatures in air, but they are much less ductile and have higher internal stress than high P alloys. However, as proven in the experiment, the latter is not an important issue in fibers coated with copper prior to nickel; hence, low P alloys seem to be more reliable for usage in high temperature sensing applications.

**Table 2:** Tensile strength of metal-coated fibers after two-step annealing, firstly at 600 °C and then after increasing the temperature to 700 °C.

Sample	Hours at 600 °C	Hours at 700 °C	Strain at breakage (mε)
Cu-coated	3	3	6.6
Cu-coated	3	3	7.6
Cu-coated + low P Ni	3	4	7.1
Cu-coated + high P Ni	3	4	3.7

When the temperature was raised to 700 °C, we noticed that the oxidation process increased drastically, even for nickel-coated fibers. We have performed a subsequent set of tests with copper-coated fibers electrochemically attached to the metal membrane. Furthermore, as high P nickel coatings showed lower temperature resistance, we have performed the tests only for two low P samples (and two pure copper-coated fibers for reference). Samples coated solely with copper withstood only 2 h in the third stage of the experiment (at 600 °C), while fibers with 5 μm-thick nickel overlayer endured 2 h more, which again proves the positive effect of electroless nickel on the fiber high-temperature resistance. Moreover, the fiber lifetime depends on the heating profile, as the time to breakage varies between the tests presented in Table 2 and Table 3.

**Table 3:** Tensile strength of metal-coated fibers after three-step annealing, firstly at 400 °C, then at 500 °C and subsequently at 600 °C.

Sample	Heating Steps			Strain at Breakage (mε)
	Hours at 400 °C	Hours at 500 °C	Hours at 600 °C	
Cu-coated	5	2	2	4.6
Cu-coated	5	2	2	7.9
Cu-coated + low P nickel	5	2	4	5.2
Cu-coated + low P nickel	5	2	4	10.5

It is important to highlight the fact that the samples in this experiment were more complex than in the previous one, since it also contained the metal membrane. Fabrication of such samples requires a series of pre-treatment processes, including etching and electroplating, which may affect the high temperature durability of the fiber, because etching decreases the coating thickness and electroplating increases it. Thus, there is a very high chance of obtaining spots with too low or too high a thickness, which will change the fiber lifetime at high temperatures. Nevertheless, this experiment provides information about the high temperature durability of optical fiber devices manufactured by electrochemical methods.

#### 3.3.4. Minimum Bending Radius Tests

**Figure 3:**
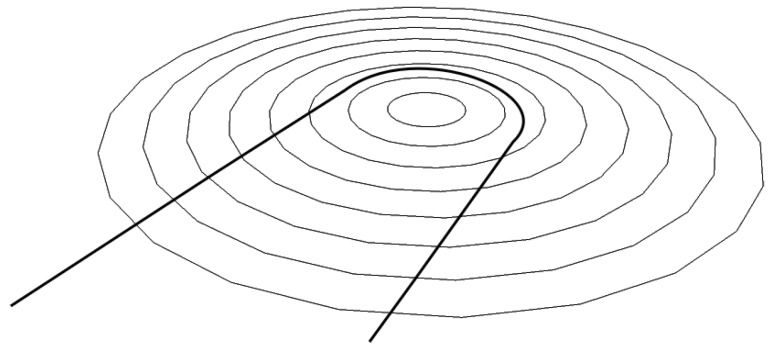
A principle of the minimum bending radius measurement.

Another way to evaluate the mechanical properties of an optical fiber in real-life sensing applications in harsh environments is to verify its transverse strain resistance, by performing a bending test. The principle of such a test is a simplified measurement technique firstly reported by Kurkjian [36,37] with the test scheme shown in Figure 3. It is noteworthy that the test refers solely to the fiber mechanical properties and should not be confused with the telecommunication fiber minimum bending radius test, at which transmission is monitored.

Results of the measurements are given in Table 4, which contains the values of the minimum bending radius before and after annealing for (1) 3 h at 500 °C and (2) 2 h at 600 °C. Each result was obtained by calculating the average from three single measurements. Beside copper- and nickel-coated fibers (with a low P nickel layer of approximately 10 μm), the results for bare fiber and acrylate-coated fiber at room temperature are given for comparison. Before annealing, a very low value of 1 mm was noted for acrylate-coated fiber, copper-coated fiber and copper-coated fiber with a nickel layer. Due to the inevitable destruction of bare and acrylate-coated fibers during annealing at high temperatures, they have not been tested for the change of minimum bending radius. After annealing at 500 °C, fibers with a nickel overlayer gave better results than fibers coated with copper only. After 2 h at 600 °C, the difference between these two fiber types got even bigger, which seems to be intuitive, since nickel serves as an additional barrier between fiber and air.

**Table 4:** Results of the minimum bending radius (MBR) tests (before annealing, 500 °C for 3 h, 600 °C for 2 h). All numbers are given in millimeters.

Sample Type	MBR Before Annealing	MBR After 3 h in 500 °C	MBR After 2 h in 600 °C
Bare	4	–	–
Acrylate-coated	1	–	–
Copper-coated	1	1.5	2
Copper-coated + 10-μm NiP	1	1	1

The positive effect of nickel coating affects not only the mechanical parameters of a fiber, the morphology after heating changes, as well, and the change is clearly visible. Samples coated with copper and copper with nickel were heated to 500 °C for 3 h, after which they looked entirely different. Electroless nickel coating after annealing is covered with a colorful oxide layer with good adhesion to the substrate, while copper-coated fibers have a black layer of copper oxides, which easily comes off the surface of the fiber.

Minimum bending radius tests give additional information about the mechanical properties of the fibers before and after the annealing. This technique has lower resolution than the tensile stress test; however, the information it provides can be particularly useful for manual operation with the optical fibers. One can use this parameter to judge if fibers can be bent before or after the heat processing. 

## 4. Extrapolation of Results to Other Temperatures

### 4.1. Basic Properties of the Extrapolation Model

The evaluation of the usefulness of metal-coated optical fibers for long-term operation at high temperatures needs verification of the possibility of extrapolating the results obtained during annealing at higher temperatures. The purpose of that is to conduct accelerated wear experiments, in which detailed knowledge of the mechanisms and kinetics of the oxidation of metals is essential.

We postulate that lifetime *t* of nickel plated copper-coated optical fibers at high temperatures can be described as:

(9) $$t=t_{\text{1}}+t_{\text{2}}+t_{\text{3}}$$

where *t*_1_ is the time needed for the degradation of the nickel layer, *t*_2_ is the time required for the degradation of the copper coating and *t*_3_ is the time needed for the degradation of the silica fiber. This model is simplified, because it does not take into account the influence of diffusion and the influence of screening the copper coating by oxidized nickel. In the first case, the discrepancy caused by omitting the diffusion is relatively small, due to the small values of interdiffusion coefficients, thanks to which nickel can be used as a barrier coating for Cu, even at high temperatures [27]. The screening effect is also insignificant due to the high porosity of the oxidized nickel layer and the relatively high rate of NiP alloy oxidation [38].

The results of only the stretching tests will be taken into account during the extrapolation. The major reason why we have chosen this technique is that it provides better resolution than the minimum bending radius test. What is more, the bending test procedure required breaking every fiber, which is inconvenient during the experiments; therefore, it must have been changed for such measurements.

Our initial experiments have shown that a bare fiber breaks at a strain within the range of 1.4–3.2 mε after annealing at 600 °C for an hour. What is more, the fiber stretching was performed with a single hour interval; thus, the lifetime of a bare silica fiber should not be taken into account in our model, since it is of the order of magnitude of the experimental uncertainty. Eventually, *t*_3_ in Equation (9) can be recognized as negligible, hence:

(10) $$t\approx t_{1}\text{+ }t_{2}$$

### 4.2. Oxidation of Metals According to the Parabolic Law

The kinetics of the metal oxidation can be described by many dependences, out of which is parabolic (Equation (11)), which is the most commonly recognized one, due to its simplicity, self-consistency and good correspondence to the experimental results [39]:

(11) $$w^{2}\text{= }kt$$

where *w* stands for weight gain per surface unit, *k* is a parabolic rate constant of oxidation and *t* is time. The oxidation rate of a sample decreases over time, due to the elongation of the route that reagents need to go through to get to the reaction site. The oxidation rate tends to continuously decrease as the oxide layer thickness increases. Furthermore, *k* changes with temperature, with the dependence given by the Arrhenius equation [39]:

(12) $$k\text{ =}\;Ae^{\frac{-E_{a}}{RT}}$$

where A is a constant (g^2^·cm^−4^·s^−1^) and *E*_a_ is activation energy (kJ/mol).

In order to extrapolate the experimental results obtained for a certain temperature to other temperatures, experimental lifetimes must be multiplied by the factor *k_T_*_1_/*k_T_*_2_, which is a consequence of dividing Equation (11) for temperature *T*_1_ by the same equation for temperature *T*_2_ (in both cases, the coating thickness is the same; thus, *w*_1_ = *w*_2_). After taking into account Equation (12), the quotient of two values of *k* at different temperatures is equal to:

(13) $$k_{T_{2}}/k_{T_{1}}{\text{= e}}^{\frac{E_{a}}{RT_{1}}-\frac{E_{a}}{RT_{2}}}$$

The results reported in [38,39,40] clearly indicate that there are different oxidation mechanisms for different temperature ranges. In each temperature range, the activation energy is different, because *E*_a_ increases with temperature. Additionally, *E*_a_ strongly depends on the metal composition. Literature data are usually given for pure metals, which are much more corrosion resistant than common alloys. Our observations indicate that copper used for fiber coating is not chemically pure, as it gets covered with a dark layer after immersion in 5% NaOH solution for a few minutes, which does not occur with pure Cu. Furthermore, metal-coated fiber samples were repeatedly stretched during our experiment, which does not correspond to the methodology reported for weight gain measurements, where no strain is applied [38,39,40]. This factor in an evident way influences the results, since the oxide layer residing on the top of the metal coating is brittle and prone to breakage during stretching. Directly underneath such fractures, oxidation takes place more vigorously, as the access to the metal surface is not hindered by the oxide layer.

Therefore, it is necessary to calculate the *E*_a_ specific for our experiments and to evaluate the whole model by checking the lifetime values for measurements from Table 2. It is important to emphasize the fact that due to the conditions of the experiments, this will not be an activation energy according to the strict definition, but an effective value of *E*_a_. It will have an approximate character, as different oxidation mechanisms may occur at different temperatures. 

### 4.3. Least Square Calculation of the Kinetic Parameters

We have performed a series of annealing experiments with concurrent stretching conducted at different temperatures, which was presented in Table 1. Thus, we can calculate the basic parameters of the parabolic equation of oxidation and apply it to our data. Taking into account Equation (11) with respect to Equation (12), one can obtain:

(14) $$\text{ln}t=\text{ln}\frac{w^{\text{2}}}{A}+\frac{E_{\text{a}}}{RT}$$

**Figure 4:**
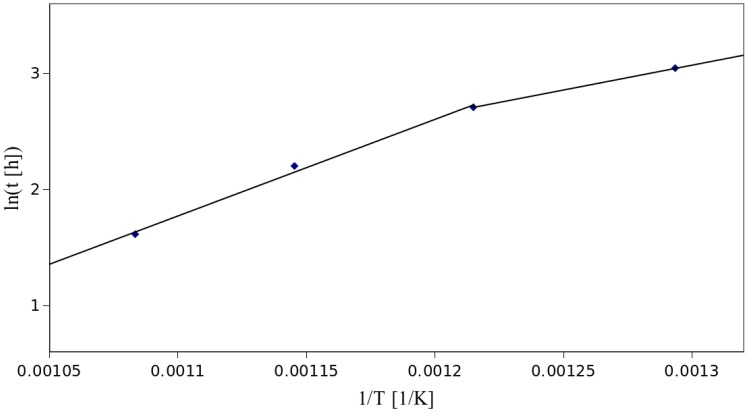
Dependence between the time of breakage and the annealing temperature of copper-coated fibers.

The above equation clearly indicates that the dependence between ln*t* and 1/*T* should be linear. In Figure 4, we have plotted these two factors.

Data plotted in Figure 4 between 550 and 650 °C are evidently linear, which is justified by the high value of *R*^2^ equal to 0.995. The resulting effective activation energy is here equal to 69.2 kJ/mol. This value is 27% lower than for 4 N copper reported for the temperature range of 550–800 °C (95 kJ) [39], which again indicates that the metal protecting the optical fiber is an alloy. Apart from that, stretching can also influence *E*_a_, and therefore, this parameter is valid only when the stretching threshold for different experiments is the same. In our case, all of the samples were stretched up to a value of 7.5 mε. The value of *A* also can be affected by stretching; however, we have not determined the exact value of this parameter, but rather, a value of *w*^2^/*A*. As was already stated, *w*^2^ depends on the coating thickness, which should be the same in every measurement. In this case, *w*^2^/*A* was equal to 6.2 × 10^−4^ g^2^·cm^−4^·s^−1^.

Similarly, we have calculated the value of effective activation energy for the data below 550 °C, which yielded 35.6 kJ/mol, while *w*^2^/*A* was equal to 8.3 × 10^−2^ g^2^·cm^−4^·s^−1^. The *E*_a_ value is 31% lower than the one reported for 4 N copper (52 kJ/mol) for the 350–550 °C range [39]. The low amount of measurements for this region exact caution during further analysis. However, the activation energy is lower than the one reported in the literature, similarly to the higher temperature range, which indicates, that this approximation should be quite accurate. What is more, other authors also report that copper exhibits different oxidation mechanisms under and above approximately 550 °C, which results in two different values of activation energy [24,39].

Data obtained during experiments with electroless nickel appear to be insufficient to determine the *E*_a_ and *w*^2^/*A*, since only two lifetime values for distant temperatures (difference of 200 °C) could be taken into account. 

### 4.4. Model Evaluation

The model described above can be evaluated by comparing the experiment from Table 2 with the time that the fiber should withstand at high temperature, according to the extrapolation model. The relative degradation of the fiber coating can be calculated with the following equation:

(15) $$\frac{w_{\text{1}}}{w_{\text{max}}}=\sqrt{e^{\frac{-E_{\text{a}}}{RT}}\cdot t}$$

where *w*_1_ stands for weight gain for a certain experiment, *w*_max_ is a maximum possible weight gain and *t* is the time of annealing since the beginning. The cylindrical shape of the coating can be neglected, because calculated values of *w*_1_/*w*_max_ only refer to the thickness of the oxidized layer, since the shape of the coating has not been taken into account.

At 600 °C, *w*_1_/*w*_max_ = 0.591 after 3 h of annealing, *i.e.*, 59.1% of the coating thickness will be oxidized. The same value of relative degradation is obtained for 1.125 h of annealing at 700 °C. After heating the fiber for two more hours at 700 °C, the relative destruction is equal to 0.985. According to our model, the whole metal layer should be oxidized to enable air to reach the silica fiber surface; therefore, one hour more at 700 °C is needed to destroy the fiber. Thus, we obtained that the fiber should break after 3 h at 600 °C and 3 h at 700 °C, which was the actual result of the experiment. Therefore, the extrapolation model can be considered reliable.

Many manufacturers provide information that copper-coated fibers can operate at 450 °C. The elaborated model can be also used for determining the lifetime of the optical fibers at this temperature. After taking into account Equation (15), one obtains 31 h of operation with repeatedly applied stress at 450 °C.

The experiment with fibers binding to the metal membrane should not be evaluated this way, due to the reasons that were already explained in Subsection 3.3.3.

## 5. Conclusions

We have presented an analysis of the long-term thermal durability of copper-coated silica fibers and a method of enhancing such durability by the deposition of an additional layer of electroless nickel-phosphorous alloy. We have experimentally verified that low phosphorus alloys are characterized by the best temperature resistance, with a significant fiber lifetime (under extreme temperature and cycled longitudinal strain) increase in comparison to sole Cu-coated fibers. We also developed a model of fiber lifetime prediction, which allows one to extrapolate the obtained results to other temperatures. The calculated effective (specific for the performed test) activation energies for Cu prove that temperature and cyclic longitudinal strain play fundamental roles in the destruction mechanism. 
